# Nivolumab in previously treated metastatic renal cell carcinoma: real-world data from the Czech Republic and Slovakia

**DOI:** 10.3389/fimmu.2026.1863009

**Published:** 2026-06-30

**Authors:** Ondřej Fiala, Michaela Tkadlecová, Tomas Buchler, Jindřich Kopecký, Zuzana Tomčová, Michal Vočka, Zuzana Syčová-Milá, Hana Študentová, Martin Matějů, Alexander Savka, Eva Šlachtová, Peter Priester, Jan Králíček, Martina Spisarová, Anežka Zemánková, Radka Lohynská, Lucie Grmelová, Jana Obertová, Petr Stránský, Bohuslav Melichar, Alexandr Poprach, Patrik Palacka

**Affiliations:** 1 Department of Oncology and Radiotherapeutics, Faculty of Medicine and University Hospital in Pilsen, Charles University, Pilsen, Czechia; 2 Biomedical Center, Faculty of Medicine in Pilsen, Charles University, Pilsen, Czechia; 3 ARON Research Foundation ETS, Macerata, Italy; 4 Department of Oncology, Second Faculty of Medicine, Charles University and Motol University Hospital, Prague, Czechia; 5 Department of Oncology, University Hospital in Hradec Králové, Hradec Králové, Czechia; 6 2^nd^ Department of Oncology, Faculty of Medicine, Comenius University, and National Cancer Institute, Bratislava, Slovakia; 7 Department of Oncology, First Faculty of Medicine, Charles University and General University Hospital, Prague, Czechia; 8 Department of Oncology, Palacký University Medical School and Teaching Hospital, Olomouc, Czechia; 9 Department of Oncology, F. D. Roosevelt University General Hospital, Banská Bystrica, Slovakia; 10 Department of Oncology, East Cancer Slovak Institute, Kosice, Slovakia; 11 Department of Oncology, First Faculty of Medicine, Charles University and Thomayer University Hospital, Prague, Czechia; 12 Department of Comprehensive Cancer Care, Masaryk Memorial Cancer Institute, Brno, Czechia; 13 Department of Comprehensive Cancer Care, Faculty of Medicine, Masaryk University, Brno, Czechia; 14 Department of Urology, Faculty of Medicine and University Hospital in Pilsen, Charles University, Pilsen, Czechia; 15 Cancer Research Institute, Biomedical Research Center of the Slovak Academy of Sciences, Bratislava, Slovakia

**Keywords:** immune checkpoint inhibitors, inflammation biomarkers, metastatic renal cell carcinoma, nivolumab, real-world data

## Abstract

**Background:**

Immune checkpoint inhibitors (ICIs) have become a cornerstone of treatment for metastatic renal cell carcinoma (mRCC). Nivolumab monotherapy remains an established option in previously treated patients; however, real-world data (RWD) from Central and Eastern Europe are limited.

**Methods:**

We conducted a retrospective multicenter cohort study using data from the RENIS II registry, including 501 patients with mRCC treated with nivolumab in the second or later line between 2013 and 2025 across the Czech Republic and Slovakia. The primary endpoints were overall survival (OS) and progression-free survival (PFS). Secondary endpoints included objective response rate (ORR), disease control rate (DCR), safety, and exploratory analyses of baseline inflammatory indices (neutrophil-to-lymphocyte ratio [NLR], platelet-to-lymphocyte ratio [PLR], and systemic immune-inflammation index [SII]).

**Results:**

Median OS was 24.2 months and median PFS was 8.6 months. The ORR was 28.8% and the DCR was 61.2% in the response-evaluable population. Grade 3/4 adverse events were recorded in 11.4% of patients. In exploratory analyses, higher baseline NLR, PLR, and SII were associated with inferior survival in univariable analyses. In multivariable models, NLR and PLR remained independently associated with OS and PFS, whereas SII did not retain independent prognostic significance.

**Conclusion:**

In this large multicenter real-world cohort, nivolumab monotherapy demonstrated consistent clinical activity and manageable toxicity in previously treated mRCC, with outcomes comparable to those reported in clinical trials and other European real-world studies. Baseline inflammatory indices, particularly NLR and PLR, showed potential prognostic value and warrant further investigation.

## Introduction

Immune checkpoint inhibitors (ICIs) have substantially reshaped the treatment landscape of metastatic renal cell carcinoma (mRCC) and are now central to contemporary systemic therapy. Within this evolving framework, nivolumab remains relevant across multiple treatment settings, from first-line combinations to later-line monotherapy after prior vascular endothelial growth factor (VEGF) tyrosine kinase inhibitors (TKIs) (1, 2). The clinical role of nivolumab monotherapy in previously treated advanced RCC was established in the phase III CheckMate 025 trial, in which nivolumab improved overall survival (OS), objective response rate (ORR), and tolerability versus everolimus (3). These benefits were maintained with extended follow-up, and nivolumab was also associated with better health-related quality of life (4, 5).

Although randomized clinical trials remain the basis for regulatory approval and guideline recommendations, real-world data (RWD) provide complementary information on treatment effectiveness, tolerability, and outcomes in broader patient populations (6). For nivolumab monotherapy in mRCC, several observational studies from routine practice settings have reported outcomes broadly consistent with the registration clinical trial (7–10). These RWD support the external validity of CheckMate 025, but they also underline the value of country- and region-specific datasets, since access to therapy and sequencing patterns may differ across the regional healthcare systems (7–10). In that context, multicenter RWD from the Czech Republic and Slovakia are of particular interest. Based on the pattern of published evidence identified above, such regional evidence appears to be less extensively reported than data from several Western European cohorts, which makes dedicated analyses from Central Europe clinically relevant (7–10).

The present retrospective multicenter study evaluates the real-world outcomes of nivolumab monotherapy in second- and later-line treatment of mRCC in patients treated across centers in the Czech Republic and Slovakia, with the aim of describing treatment effectiveness and providing a region-specific benchmark for everyday clinical practice.

## Patients and methods

### Study design and population

The present retrospective multicenter real-world cohort study evaluated the outcomes of nivolumab monotherapy in patients with mRCC treated in routine clinical practice between 2013 and 2025. Eligible patients were adults with histologically confirmed mRCC who received nivolumab in the second or later line of systemic treatment after prior therapy. None of the patients had received prior ICI therapy. The RWD from seven cancer centers in the Czech Republic and three cancer centers in the Slovakia were analyzed. Clinical data were obtained from the Renal Cell Carcinoma Information System II (RENIS II) registry (http://renis.registry.cz), which has been described previously (11).

### Study objectives

The primary aim of the present study was to describe the real-world clinical outcomes of nivolumab monotherapy in second- and later-line treatment of mRCC. The main time-to-event endpoints were progression-free survival (PFS) and overall survival (OS). Tumor response outcomes included objective response rate (ORR), defined as the proportion of patients achieving complete response (CR) or partial response (PR) as best overall response, and disease control rate (DCR), defined as the proportion achieving CR, PR, or stable disease (SD).

The secondary aims included survival outcomes in specific patient populations, safety with focus on grade 3/4 adverse events (AEs), and exploratory biomarker evaluation including baseline neutrophil-to-lymphocyte ratio (NLR), platelet-to-lymphocyte ratio (PLR), and systemic immune-inflammation index (SII) derived from routine blood counts recorded at nivolumab initiation. NLR was calculated as the absolute neutrophil count divided by the absolute lymphocyte count, PLR as the platelet count divided by the absolute lymphocyte count, and SII as platelet count × absolute neutrophil count/absolute lymphocyte count. These inflammatory indices were selected based on previously reported prognostic associations in mRCC, including studies in patients treated with PD-1 blockade and immune-based therapies (12–20).

### Treatment and follow-up

Patients received nivolumab monotherapy according to local clinical practice and the applicable standard of care during the study period. Treatment was continued until disease progression, unacceptable toxicity, death, or physician/patient decision. The follow-up visits including physical examination and routine laboratory tests were performed every two to four weeks, and computed tomography (CT) was performed every three to four months during the treatment, the results were assessed locally using Response Evaluation Criteria in Solid Tumors (RECIST) version 1.1 (21). Treatment-related adverse events (AEs) were assessed retrospectively from the medical records and graded according to the Common Terminology Criteria for Adverse Events (CTCAE), version 5.0 (22).

### Statistical analysis

Baseline characteristics were summarized using standard descriptive statistics. Continuous variables were reported as median with interquartile range (IQR) or mean with standard deviation (SD), as appropriate, and categorical variables as counts and percentages. PFS was defined as the time from nivolumab initiation to documented disease progression or death from any cause, whichever occurred first. OS was defined as the time from nivolumab initiation to death from any cause. Patients without an event at the time of data cut-off were censored at the date of last available follow-up. Survival distributions were estimated using the Kaplan-Meier method and compared using the log-rank test. Median survival times with 95% confidence intervals (CIs) were reported. Hazard ratios (HRs) with 95% CIs for PFS and OS were estimated using Cox proportional hazards regression models. Univariable analyses were performed for selected baseline clinicopathological variables. Multivariable Cox models were constructed to assess the independent association of selected variables with survival outcomes, with covariates selected according to clinical relevance, biological plausibility, and completeness of available data rather than by purely data-driven variable screening. For exploratory biomarker analyses, NLR, PLR, and SII were analyzed as categorical variables using cohort-specific median values as cut-offs. Median-based thresholds were selected because universally accepted clinically validated cut-offs for nivolumab-treated mRCC populations are lacking and to avoid outcome-driven optimization approaches that could increase the risk of overfitting. Kaplan-Meier analyses and log-rank tests were used to compare PFS and OS between patients with values below versus above the median. Biomarker analyses were considered exploratory and hypothesis-generating. Kaplan-Meier analyses and log-rank tests were used to compare PFS and OS between patients with values below versus above the median. Missing data were not imputed, and analyses were performed on available cases. All statistical tests were two-sided, and a p value <0.05 was considered statistically significant. No formal adjustment for multiple testing was applied. Statistical analyses were performed in Python 3.13.5 using pandas 2.2.3 and NumPy 2.3.5 for data processing and variable derivation, statsmodels 0.14.6 for Kaplan-Meier estimation, log-rank testing, and Cox proportional hazards modeling, SciPy 1.17.0 for categorical comparisons, and matplotlib 3.10.8 for figure generation.

## Results

### Patient population

A total of 501 mRCC patients were included in the present study. Baseline characteristics of the study population are summarized in **Table 1**. Nivolumab was administered predominantly in the second and third treatment lines. Specifically, 312 patients (62.3%) received nivolumab in the second line, 172 (34.3%) in the third line, 14 (2.8%) in the fourth line, and 3 (0.6%) in the fifth line.

**Table 1:** Baseline characteristics of the treated cohort overall and according to line of nivolumab therapy.

Characteristic	Overall (n=501)	2L (n=312)	3L+ (n=189)
Median age at nivolumab initiation, years (IQR)	67.4 (60.6–72.9)	67.7 (60.5–73.5)	66.5 (60.6–72.0)
Sex			
Male	368 (73.5)	225 (72.1)	143 (75.7)
Female	133 (26.5)	87 (27.9)	46 (24.3)
ECOG PS			
0	154 (30.7)	100 (32.1)	54 (28.6)
1	285 (56.9)	173 (55.4)	112 (59.3)
2	29 (5.8)	19 (6.1)	10 (5.3)
Not available	33 (6.6)	20 (6.4)	13 (6.9)
Synchronous metastases			
Yes	188 (37.5)	129 (41.3)	59 (31.2)
No	285 (56.9)	162 (51.9)	123 (65.1)
Not available	28 (5.6)	21 (6.7)	7 (3.7)
Bone metastases			
Yes	100 (20.0)	59 (18.9)	41 (21.7)
No	401 (80.0)	253 (81.1)	148 (78.3)
Grade			
1	48 (9.6)	28 (9.0)	20 (10.6)
2	144 (28.7)	84 (26.9)	60 (31.7)
3–4	229 (45.7)	149 (47.8)	80 (42.3)
Not applicable	69 (13.8)	45 (14.4)	24 (12.7)
Not available	11 (2.2)	6 (1.9)	5 (2.6)
Histology			
Clear-cell	335 (66.9)	210 (67.3)	125 (66.1)
Papillary	18 (3.6)	10 (3.2)	8 (4.2)
Other non-clear-cell	14 (2.8)	10 (3.2)	4 (2.1)
Not available	134 (26.7)	82 (26.3)	52 (27.5)
Previous nephrectomy			
Yes	412 (82.2)	241 (77.2)	171 (90.5)
No	89 (17.8)	71 (22.8)	18 (9.5)
IMDC risk group			
Favorable	134 (26.7)	82 (26.3)	52 (27.5)
Intermediate	247 (49.3)	162 (51.9)	85 (45.0)
Poor	57 (11.4)	32 (10.3)	25 (13.2)
Not available	63 (12.6)	36 (11.5)	27 (14.3)
Baseline NLR			
Median (IQR)	2.85 (1.86–4.22)	2.60 (1.74–3.94)	3.02 (2.03–4.43)
Baseline PLR			
Median (IQR)	165.1 (111.3–253.7)	163.7 (108.4–258.2)	166.7 (119.4–245.4)
Baseline SII			
Median (IQR)	697.9 (412.3–1204.4)	654.9 (382.5–1176.7)	775.6 (473.6–1274.6)

### Survival outcomes and objective response

**Figure 1:**
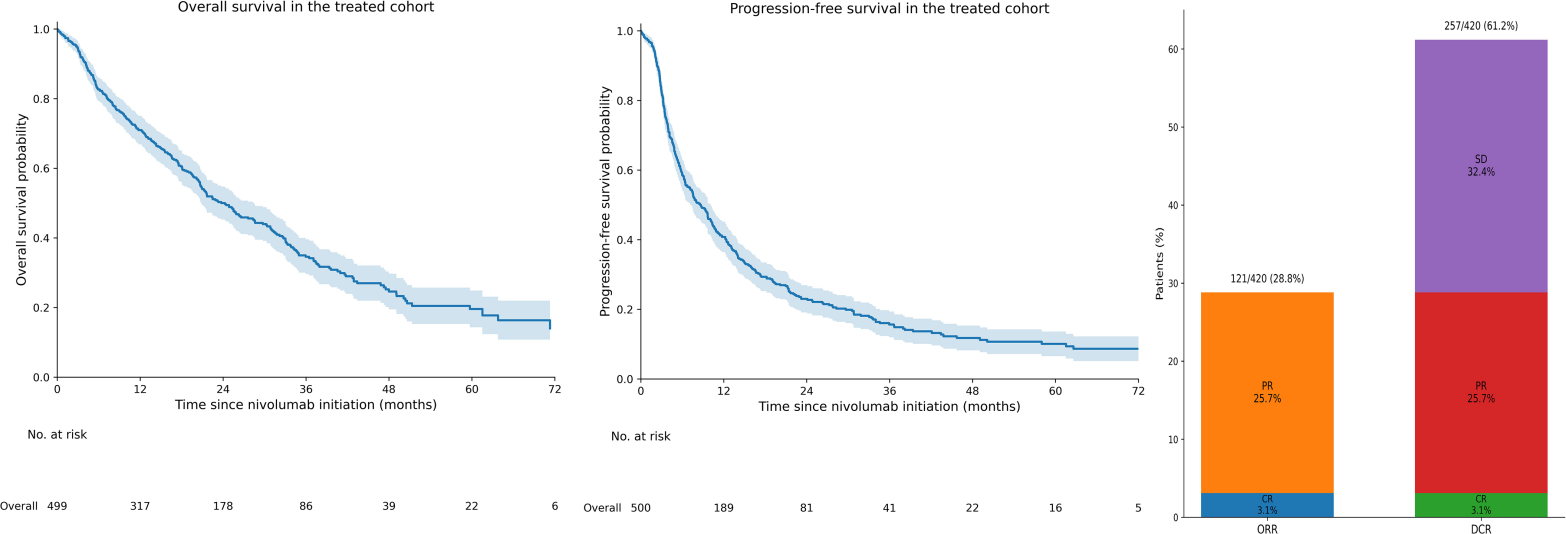
Overall survival (OS), progression-free survival (PFS), objective response rate (ORR), and disease control rate (DCR) in the overall population treated with nivolumab monotherapy.

For survival analyses, 499 patients were evaluable for OS and 500 patients for PFS after exclusion of records with missing or chronologically inconsistent follow-up information. The median follow-up was 35.8 months (95% CI, 32.9–40.5). Median OS was 24.2 months, and median PFS was 8.6 months (**Figure 1**). The estimated 12-month and 24-month OS rates were 71.0% and 50.0%, whereas the estimated 6-month and 12-month PFS rates were 58.5% and 40.8%, respectively.

A total of 420 patients had a recorded best overall response. Among the response-evaluable population, the best overall response was CR in 13 patients (3.1%), PR in 108 patients (25.7%), SD in 136 patients (32.4%), and PD in 163 patients (38.8%). The ORR was 28.8% (121/420), and the DCR was 61.2% (257/420) (**Figure 1**; **Table 2**).

**Table 2:** Treatment exposure, tumor response, and grade 3/4 adverse events in the overall cohort and according to line of nivolumab therapy.

Characteristic	Overall (n=501)	2L (n=312)	3L+ (n=189)
Response-evaluable population, n	420	262	158
Complete response (CR), n (%)	13 (3.1)	8 (3.1)	5 (3.2)
Partial response (PR), n (%)	108 (25.7)	73 (27.9)	35 (22.2)
Stable disease (SD), n (%)	136 (32.4)	84 (32.1)	52 (32.9)
Progressive disease (PD), n (%)	163 (38.8)	97 (37.0)	66 (41.8)
Objective response rate (ORR), n (%)	121 (28.8)	81 (30.9)	40 (25.3)
Disease control rate (DCR), n (%)	257 (61.2)	165 (63.0)	92 (58.2)
Grade 3/4 adverse events, n (%)	57 (11.4)	39 (12.5)	18 (9.5)
Line of nivolumab therapy, n (%)	—	312 (62.3)	189 (37.7)

### Subgroup survival outcomes

**Figure 2:**
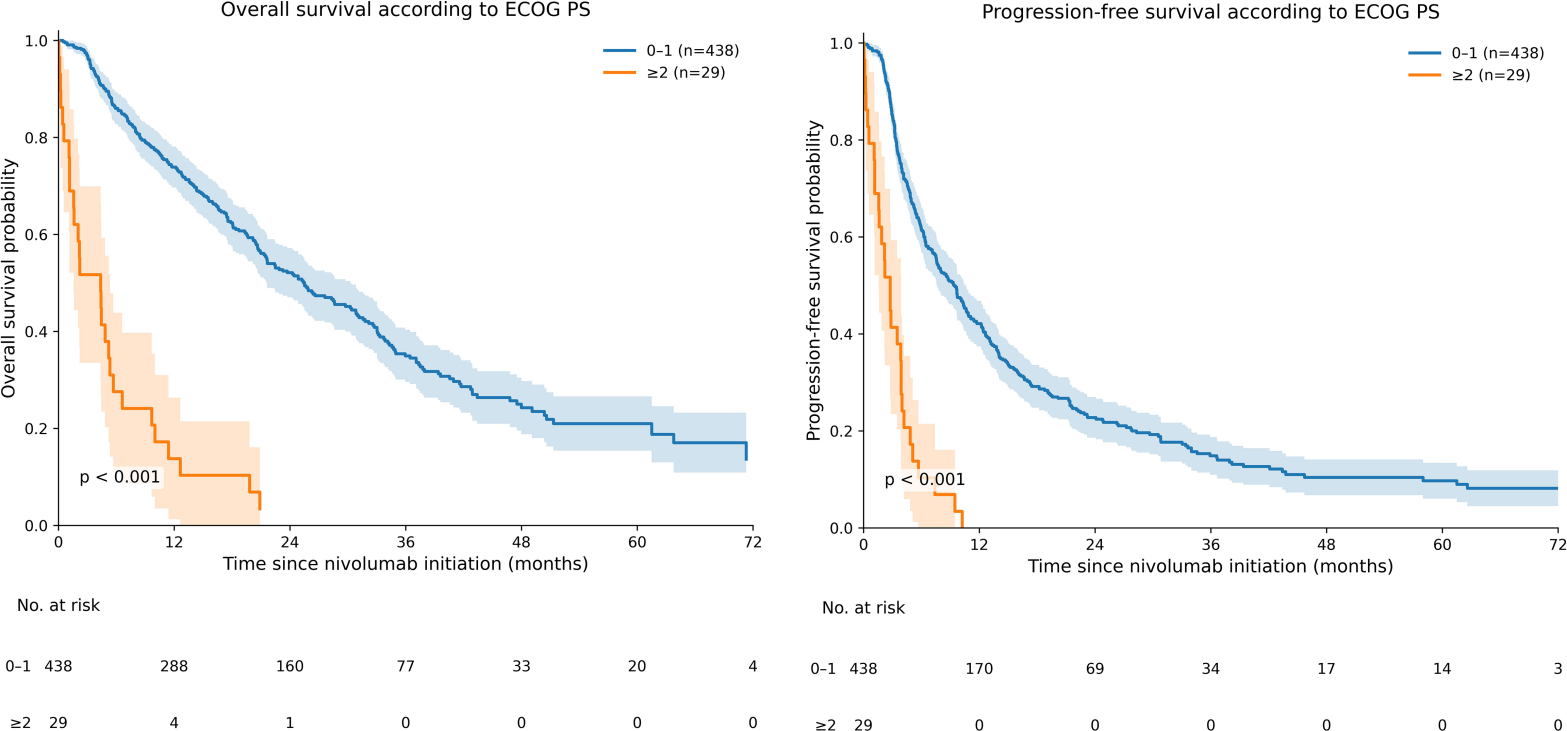
Overall survival (OS) and progression-free survival (PFS) according to ECOG PS.

Survival outcomes differed across several prespecified clinical subgroups. Patients with ECOG PS 0–1 had substantially longer OS and PFS than those with ECOG PS ≥2 (25.5 vs 4.4 months and 9.4 vs 2.8 months; both p<0.001) (**Figure 2**). Better outcomes were also observed in patients with prior nephrectomy (OS 26.4 vs 12.7 months, p<0.001; PFS 9.7 vs 4.8 months, p=0.002) (**Supplementary Figure S1**), without bone metastases (OS 28.3 vs 18.1 months, p=0.0018; PFS 9.5 vs 6.2 months, p=0.0022) (**Supplementary Figure S2**), and with metachronous rather than synchronous metastatic disease (OS 28.6 vs 17.5 months, p<0.001; PFS 9.7 vs 6.5 months, p=0.0119) (**Supplementary Figure S3**). Clear-cell histology was associated with longer OS than non-clear-cell RCC (24.4 vs 15.5 months, p=0.0034), whereas PFS did not differ significantly (7.8 vs 6.2 months, p=0.202) (**Supplementary Figure S4**). IMDC risk groups also showed a marked gradient, with median OS of 31.3, 25.5, and 5.7 months and median PFS of 7.5, 10.4, and 3.5 months in the favorable-, intermediate-, and poor-risk groups, respectively (both p<0.001) (**Supplementary Figure S5**). No meaningful differences were observed according to treatment line or age group. Detailed subgroup outcomes are summarized in **Table 3**.

**Table 3:** Overall survival and progression-free survival according to prespecified clinical subgroups.

Subgroup	n	Median OS,	Log-rank p value	Median PFS,	Log-rank p value
		months	(OS)	months	(PFS)
Line of nivolumab therapy					
2L	312	23.2	0.933	08.7	0.686
3L+	189	24.8		8.0	
ECOG PS					
0–1	439	25.5	<0.001	09.4	<0.001
≥2	29	04.4		02.8	
Nephrectomy					
Yes	412	26.4	<0.001	09.7	0.002
No	89	12.7		04.8	
Bone metastases					
No	401	28.3	0.0018	09.5	0.0022
Yes	100	18.1		06.2	
Synchronous metastatic disease					
No	285	28.6	<0.001	09.7	0.0119
Yes	188	17.5		06.5	
Age group, years					
<70	317	25.7	0.531	08.3	0.701
≥70	183	21.3		09.5	
Histology					
Clear-cell	335	24.4	0.0034	07.8	0.202
Non-clear-cell	32	15.5		06.2	
IMDC risk group					
Favorable	134	31.3	<0.001	07.5	<0.001
Intermediate	247	25.5		10.4	
Poor	57	05.7		03.5	

### Multivariable analyses

In multivariable Cox regression adjusted for prespecified clinical covariates, ECOG PS ≥2, absence of previous nephrectomy, and IMDC poor risk remained independently associated with shorter OS. The strongest association was observed for ECOG PS ≥2 (HR 4.32, 95% CI 2.66–7.00; p<0.001), followed by absence of nephrectomy (HR 1.62, 95% CI 1.15–2.29; p=0.006) and IMDC poor risk (HR 2.11, 95% CI 1.39–3.20; p<0.001). For PFS, ECOG PS ≥2 (HR 4.43, 95% CI 2.74–7.18; p<0.001), absence of nephrectomy (HR 1.51, 95% CI 1.10–2.06; p=0.010), bone metastases (HR 1.37, 95% CI 1.05–1.79; p=0.020), and IMDC poor risk (HR 1.59, 95% CI 1.08–2.36; p=0.019) remained independently associated with shorter PFS. Detailed results of the multivariable models are shown in **Table 4**.

**Table 4:** Multivariable Cox regression analyses for overall survival and progression-free survival.

	Overall survival (OS)			Progression-free survival (PFS)		
Variable	HR	95% CI	p value	HR	95% CI	p value
Age (per year)	1.00	0.99–1.01	0.756	1.00	0.99–1.01	0.493
Male sex	1.17	0.88–1.55	0.277	0.84	0.67–1.07	0.154
ECOG PS ≥2 vs 0–1	4.32	2.66–7.00	<0.001	4.43	2.74–7.18	<0.001
Line of therapy 3L+ vs 2L	01.7	0.83–1.38	0.620	01.10	0.88–1.37	0.412
Previous nephrectomy no vs yes	1.62	1.15–2.29	0.006	1.51	1.10–2.06	0.010
Bone metastases yes vs no	1.26	0.93–1.70	0.135	1.37	1.05–1.79	0.020
Synchronous metastatic disease yes vs no	1.13	0.85–1.51	0.394	01.11	0.87–1.42	0.380
Non-clear-cell vs clear-cell	1.19	0.73–1.95	0.491	0.91	0.58–1.45	0.701
Missing/unknown histology vs clear-cell	0.83	0.62–1.11	0.202	0.70	0.53–0.91	0.007
IMDC poor vs favorable/intermediate	02.11	1.39–3.20	<0.001	1.59	1.08–2.36	0.019

### Safety

**Figure 3:**
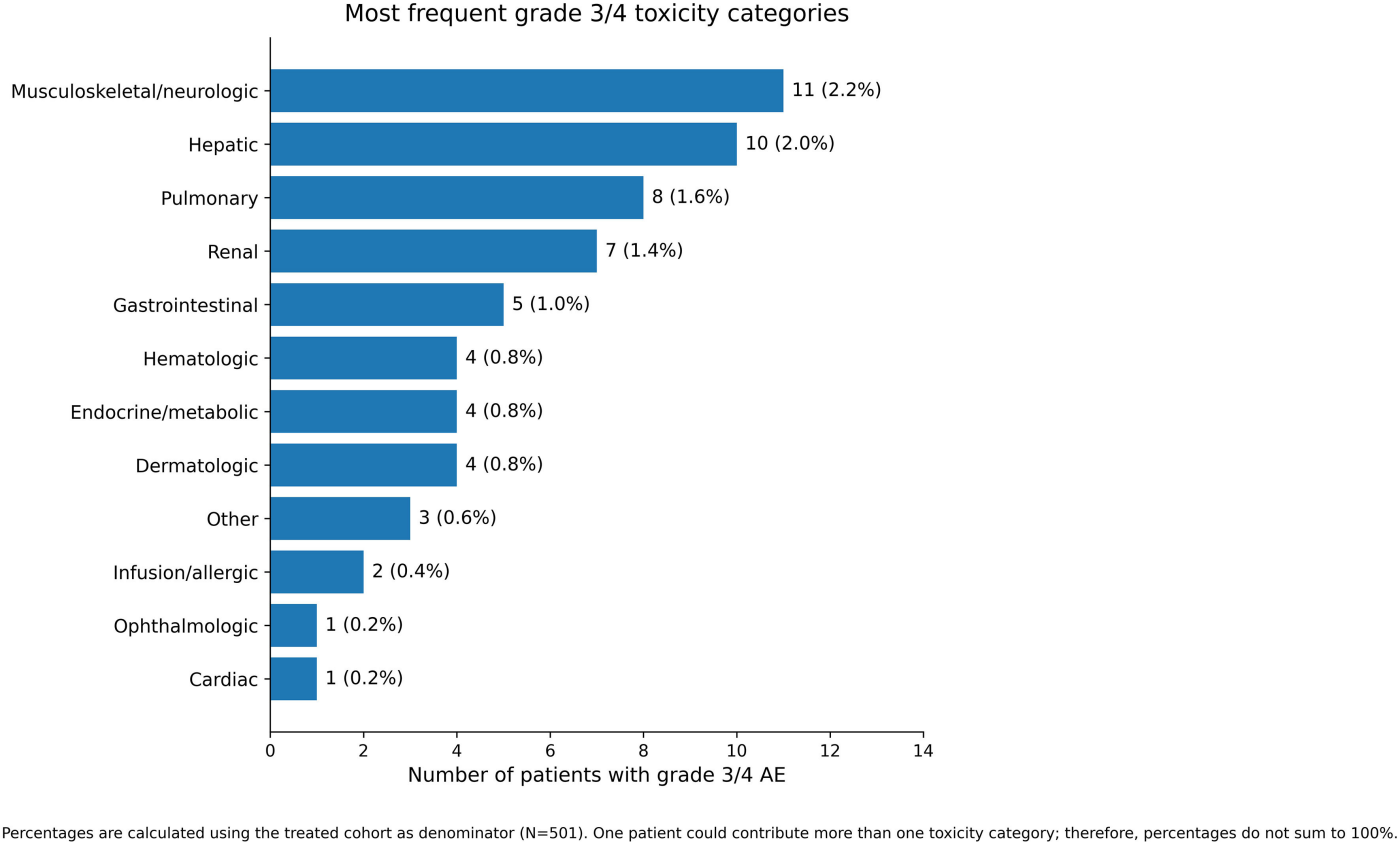
Incidence and type of grade 3/4 treatment-related adverse effects (AEs).

Grade 3/4 AEs were recorded in 57 of 501 patients (11.4%). The recorded grade 3/4 AEs were clinically heterogeneous, with the most common being musculoskeletal/neurological, hepatic, pulmonary, renal, endocrine and dermatological disorders. The incidence and type of grade 3/4 AEs are shown in **Figure 3**.

### Baseline inflammatory indices

**Figure 4:**
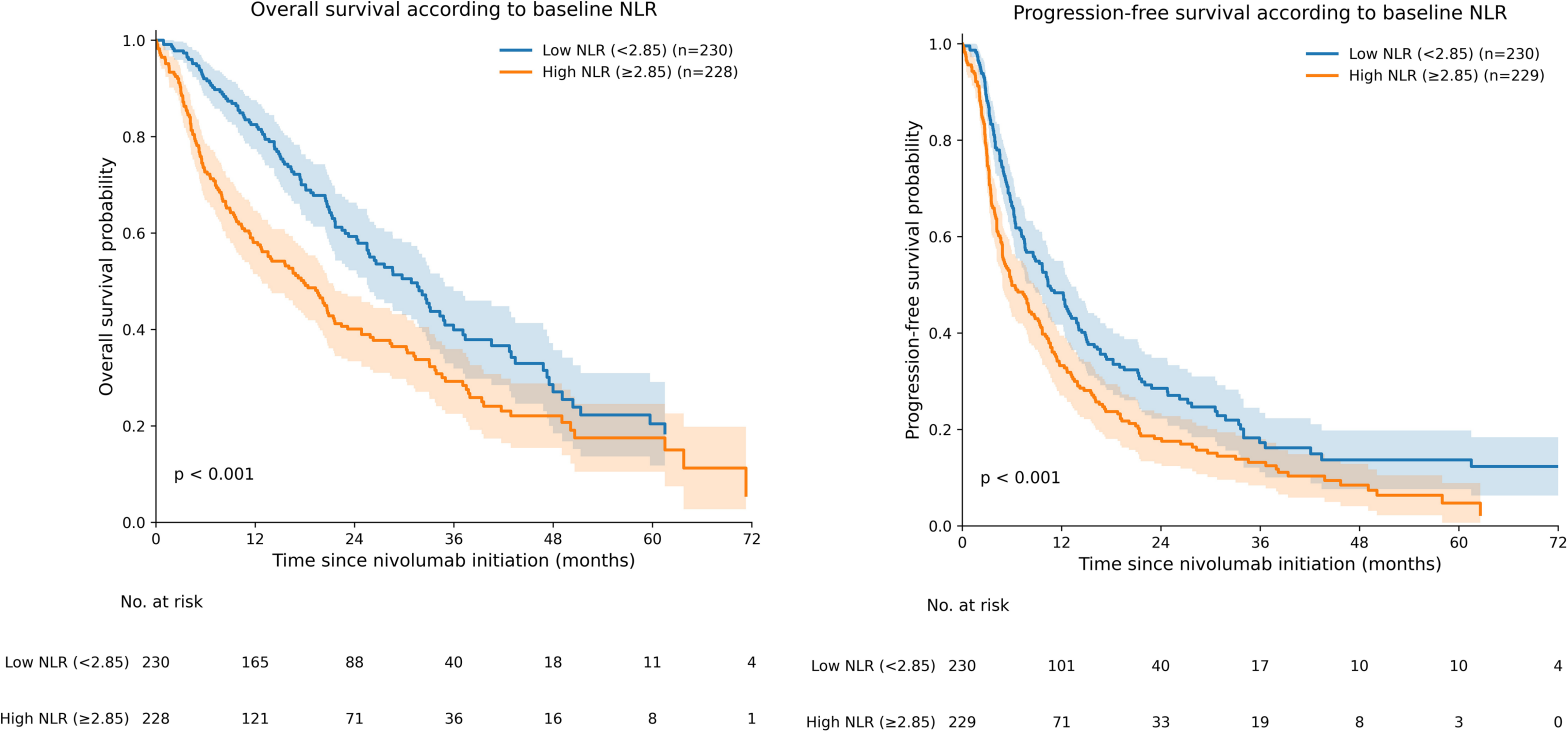
Overall survival (OS) and progression-free survival (PFS) according to baseline neutrophil-to-lymphocyte ratio (NLR).

**Figure 5:**
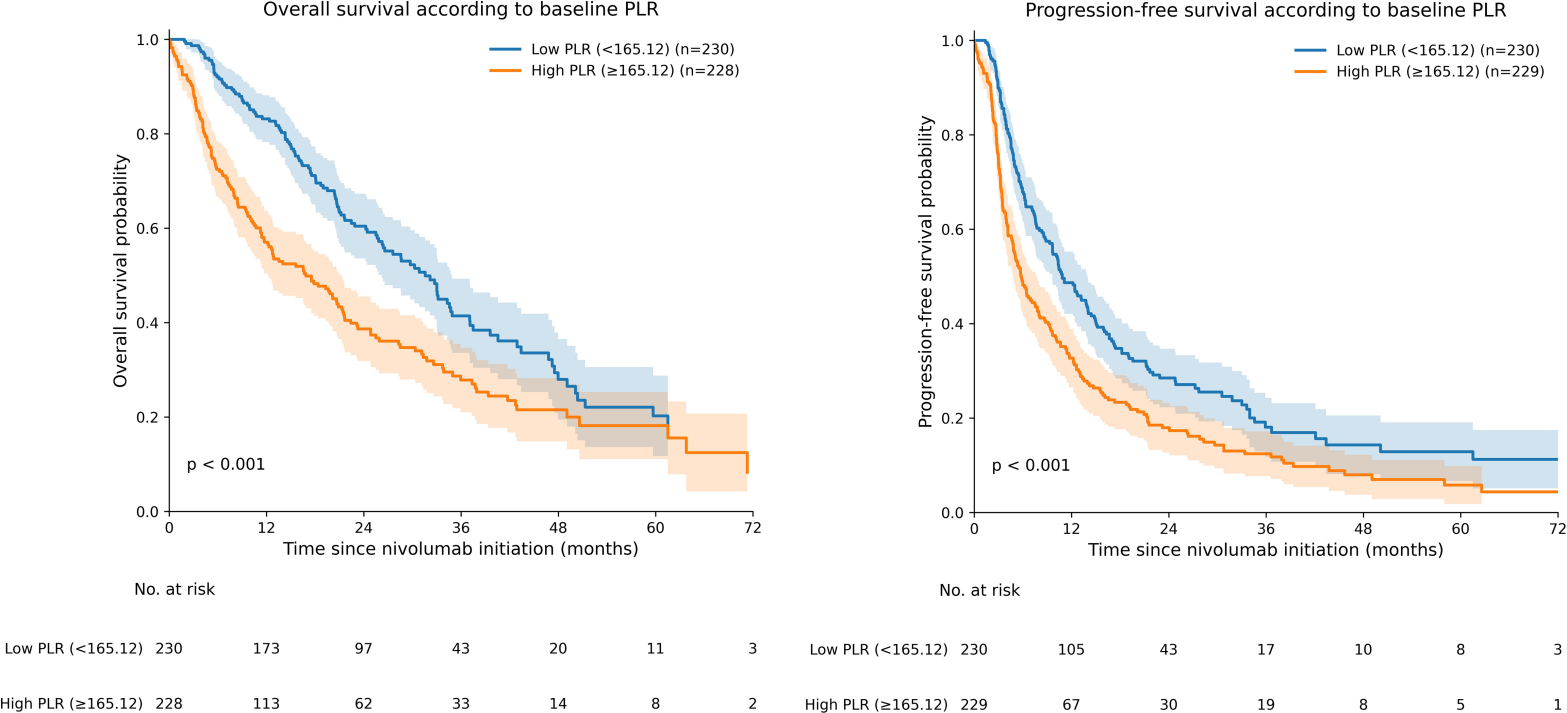
Overall survival (OS) and progression-free survival (PFS) according to baseline platelet-to-lymphocyte ratio (PLR).

In exploratory analyses using median-based cut-offs, baseline SII, NLR, and PLR were each associated with inferior survival in unadjusted analyses. Patients with high SII had shorter OS (18.4 vs 28.6 months; p=0.002) and PFS (5.7 vs 10.7 months; p=0.002) than those with low SII (**Supplementary Figure S6**). Similarly, high NLR was associated with inferior OS (17.8 vs 30.9 months; p<0.001) and PFS (6.0 vs 10.3 months; p<0.001) (**Figure 4**), while high PLR was associated with inferior OS (16.9 vs 31.6 months; p<0.001) and PFS (5.7 vs 10.8 months; p<0.001) (**Figure 5**). In multivariable Cox models adjusted for prespecified clinical covariates, high baseline NLR remained independently associated with inferior OS (HR 1.33, 95% CI 1.02–1.74; p=0.038) and PFS (HR 1.40, 95% CI 1.11–1.77; p=0.005) (**Supplementary Table S1**). Likewise, high baseline PLR remained independently associated with inferior OS (HR 1.34, 95% CI 1.01–1.76; p=0.040) and PFS (HR 1.29, 95% CI 1.02–1.63; p=0.033) (**Supplementary Table S2**). By contrast, although high baseline SII was associated with worse outcomes in unadjusted analyses, it did not retain independent prognostic significance after multivariable adjustment (OS: HR 1.10, 95% CI 0.84–1.44; p=0.483; PFS: HR 1.21, 95% CI 0.96–1.52; p=0.115) (**Supplementary Table S3**).

## Discussion

In the present retrospective multicenter real-world cohort from the Czech Republic and Slovakia, we evaluated nivolumab monotherapy in 501 mRCC patients treated in the second or later line. In the evaluable response population, the ORR was 28.8% and the DCR was 61.2%. Median PFS was 8.6 months and median OS was 24.2 months, while grade 3/4 treatment-related AEs were recorded in 11.4% of patients. Taken together, these findings support the real-world clinical activity and manageable tolerability of nivolumab monotherapy in previously treated mRCC.

The efficacy of nivolumab monotherapy in previously treated mRCC was established in CheckMate 025, in which nivolumab improved OS versus everolimus, with a median OS of 25.0 months, median PFS of 4.6 months, and an ORR of 25% (3). Long-term follow-up confirmed maintained survival benefit, durable responses, and no new safety signals (4, 5). In that context, the outcomes observed in the present real-world cohort appear broadly consistent with the registrational randomized trial. The present study median OS of 24.2 months was very close to that reported in CheckMate 025, whereas the ORR of 28.8% was numerically slightly higher. By contrast, the present PFS of 8.6 months exceeded that of CheckMate 025, which likely reflects differences in patient selection, event capture, assessment frequency, and the known limitations of the present study rather than a true superiority signal. The present study results also compare favorably with previously published European real-world studies of nivolumab monotherapy. In the German prospective noninterventional NORA study, which included 228 patients, median OS was 24.0 months, median PFS was 5.3 months, and ORR was 20% (7). In the Dutch multicenter registry-based study of 264 patients, median OS was 18.7 months, ORR was 18.6%, and grade 3/4 AEs occurred in 15% of patients (8). In the retrospective cohort from Croatia, Hungary, and Malta, which included 87 patients treated in the second or later line, median OS was 18.0 months, median PFS was 8.5 months, and grade 3/4 AEs were reported in 5% of patients (10). More recently, the Spanish Genitourinary Oncology Group reported outcomes from a multicenter retrospective cohort of 222 previously treated mRCC patients, with median OS of 18.1 months, median PFS of 4.96 months, DCR of 51%, and ORR of 23% (23). Against this background, the present cohort showed numerically favorable efficacy outcomes, particularly for ORR, DCR, and OS, while maintaining a toxicity profile within the range reported across prior real-world series. However, such cross-study comparisons should be interpreted with caution, given variation in IMDC distribution, prior treatment exposure, response-evaluable populations, follow-up duration, and local routine-practice patterns. The recently published Spanish study is particularly relevant because it provides a contemporary multicenter European benchmark in a population of previously treated patients managed in routine care (23). Their median OS of 18.1 months and median PFS of 4.96 months were shorter than those observed in the present study, while ORR and DCR were also lower at 23% and 51%, respectively (23). One plausible explanation is case-mix heterogeneity between cohorts, including differences in prognostic risk distribution, nephrectomy status, prior lines, and response assessment practices.

The observed toxicity profile should also be interpreted in light of the retrospective design. Grade 3/4 AEs were recorded in 11.4% of patients with available toxicity data, which is numerically lower than the 19% rate of grade 3/4 treatment-related AEs reported with nivolumab in CheckMate 025 and somewhat lower than the 15% rate of grade 3/4 immune-related adverse events described in the Dutch registry, while remaining higher than the 5% reported in the smaller Croatia/Hungary/Malta cohort (3, 8, 10). Such differences are expected, since retrospective real-world toxicity capture is generally less complete than prospective trial reporting.

Chronic inflammation is a hallmark of cancer and contributes to tumor progression throughout the disease course (24, 25). Cancer–immune system interactions are complex, and inflammatory blood cells such as neutrophils, platelets, and lymphocytes can shape tumor behavior (26). Accordingly, peripheral blood–derived inflammatory indices, including NLR, PLR, and SII, have emerged as potential prognostic markers in mRCC (12–20). These composite indices are generally considered to better reflect the balance between systemic inflammation and host immune status than isolated hematologic parameters alone.

The exploratory analyses of baseline inflammatory indices in the present study show that higher baseline NLR, PLR, and SII were associated with inferior PFS and OS, while multivariable Cox models revealed that high baseline NLR and high baseline PLR remained independently associated with inferior OS and PFS. These findings are consistent with previous studies that identified NLR, PLR, and related inflammatory indices as adverse prognostic factors in mRCC, including cohorts treated with nivolumab or immune-based therapies (12–19). In the present study, biomarker analyses were exploratory and used cohort-specific median cut-offs because universally accepted clinically validated thresholds for nivolumab-treated mRCC populations are lacking. In addition, outcome-driven optimization approaches were intentionally avoided in order to reduce the risk of overfitting. Accordingly, these findings should be interpreted as exploratory signals requiring prospective validation before clinical implementation or treatment decision-making.

This study has several limitations. First, its retrospective design introduces the risk of selection bias, information bias, and residual confounding. Second, response assessment, toxicity reporting, and follow-up schedules were not standardized across centers. Third, some variables were incompletely available, and missing data were not imputed. Fourth, treatment-related adverse events were collected retrospectively and were clinically heterogeneous, which limits the robustness of exploratory association analyses between toxicity, survival outcomes, and inflammatory biomarkers. Fifth, biomarker analyses were exploratory and no formal adjustment for multiple testing was applied. Although histological subgroup analyses were performed, dedicated biomarker analyses within individual histological subsets were not performed because the non-clear-cell subgroup was relatively small and clinically heterogeneous. Finally, because the cohort reflects a treatment era in which nivolumab monotherapy had a larger role in previously treated disease, the direct generalizability of these findings to the current frontline combination era is limited. Despite these limitations, the study also has notable strengths, including its multicenter design, large regional cohort, and focus on a clinically well-defined population receiving nivolumab monotherapy after prior systemic treatment. In addition, the use of routinely collected laboratory, response, survival, and toxicity data enhances the relevance of the findings for everyday clinical practice.

## Conclusions

The present multicenter real-world analysis supports the clinical activity and manageable tolerability of nivolumab monotherapy in second- and later-line mRCC. Compared with previously reported European routine-practice cohorts, the present RWD showed numerically favorable survival and response outcomes while maintaining a safety profile consistent with established experience (4–7). Exploratory analyses of baseline inflammatory indices suggest a potential prognostic role of NLR and PLR.

## Data Availability

The raw data supporting the conclusions of this article will be made available by the authors, without undue reservation.
