# Supplementary material for: Nivolumab in previously treated metastatic renal cell carcinoma: real-world data from the Czech Republic and Slovakia
Source: Front Immunol. 2026 Jun 30;17:1863009. doi: 10.3389/fimmu.2026.1863009 (PMC13364914; doi:10.3389/fimmu.2026.1863009)
Supplement: Supplementary file 1 [file SupplementaryFile1.docx]

**SUPPLEMENTARY DATA**

**Nivolumab in Previously Treated Metastatic Renal Cell Carcinoma: Real-World Data From the Czech Republic and Slovakia**

Ondřej Fiala^1,2,3^*^§^, Michaela Tkadlecová^1,2^*, Tomas Buchler^4^, Jindřich Kopecký^5^, Zuzana Tomčová^6^, Michal Vočka^7^, Zuzana Syčová-Milá^6^, Hana Študentová^8^, Martin Matějů^7^, Alexander Savka^9^, Eva Šlachtová^10^, Peter Priester^7^, Jan Králíček^1^, Martina Spisarová^8^, Anežka Zemánková^8^, Radka Lohynská^11^, Lucie Grmelová^12,13^, Jana Obertová^6^, Petr Stránský jr.^14^, Bohuslav Melichar^8^, Alexandr Poprach^12,13#^, Patrik Palacka^6,15#^

^1^Department of Oncology and Radiotherapeutics, Faculty of Medicine and University Hospital in Pilsen, Charles University, Czech Republic , alej Svobody 80, 304 60 Pilsen, Czech Republic

^2^Biomedical Center, Faculty of Medicine in Pilsen, Charles University, alej Svobody 76, 304 60 Pilsen, Czech Republic

^3^ARON Research Foundation ETS, Macerata, Italy

^4^Department of Oncology, Second Faculty of Medicine, Charles University and Motol University Hospital, V Úvalu 84, 150 06 Prague, Czech Republic

^5^Department of Oncology, University Hospital in Hradec Králové, Sokolská 581, Hradec Králové 50005, Czech Republic

^6^2^nd^ Department of Oncology, Faculty of Medicine, Comenius University, and National Cancer Institute, Bratislava, Slovakia

^7^Department of Oncology, First Faculty of Medicine , Charles University and General University Hospital, U Nemocnice 499/2, 128 08 Prague, Czech Republic

^8^Department of Oncology, Palacký University Medical School and Teaching Hospital, I.P. Pavlova 6, Olomouc, 775 20, Czech Republic.

^9^Department of Oncology, F. D. Roosevelt University General Hospital, Banská Bystrica, Slovakia

^10^Department of Oncology, East Cancer Slovak Institute, Kosice, Slovakia

^11^Department of Oncology, First Faculty of Medicine, Charles University and Thomayer University Hospital, Videnska 800, 140 59 Prague, Czech Republic

^12^Department of Comprehensive Cancer Care, Masaryk Memorial Cancer Institute, Zluty kopec 7, 656 53 Brno, Czech Republic

^13^Department of Comprehensive Cancer Care, Faculty of Medicine, Masaryk University, Kamenice 5, 625 00 Brno, Czech Republic

^14^Department of Urology, Faculty of Medicine and University Hospital in Pilsen, Charles University, Czech Republic , Edvarda Beneše 1128/13, 301 00 Pilsen, Czech Republic

^15^Cancer Research Institute, Biomedical Research Center of the Slovak Academy of Sciences, Bratislava, Slovakia.

* contributed equally as first authors

**#** contributed equally as co-senior authors

**^§^ Corresponding author**: Ondřej Fiala, M.D., Ph.D., Department of Oncology and Radiotherapeutics, Faculty of Medicine and University Hospital in Pilsen, Charles University, Czech Republic , alej Svobody 80, 304 60 Pilsen, Czech Republic. Tel.: +42 0728655488, e-mail: fialao@fnplzen.cz


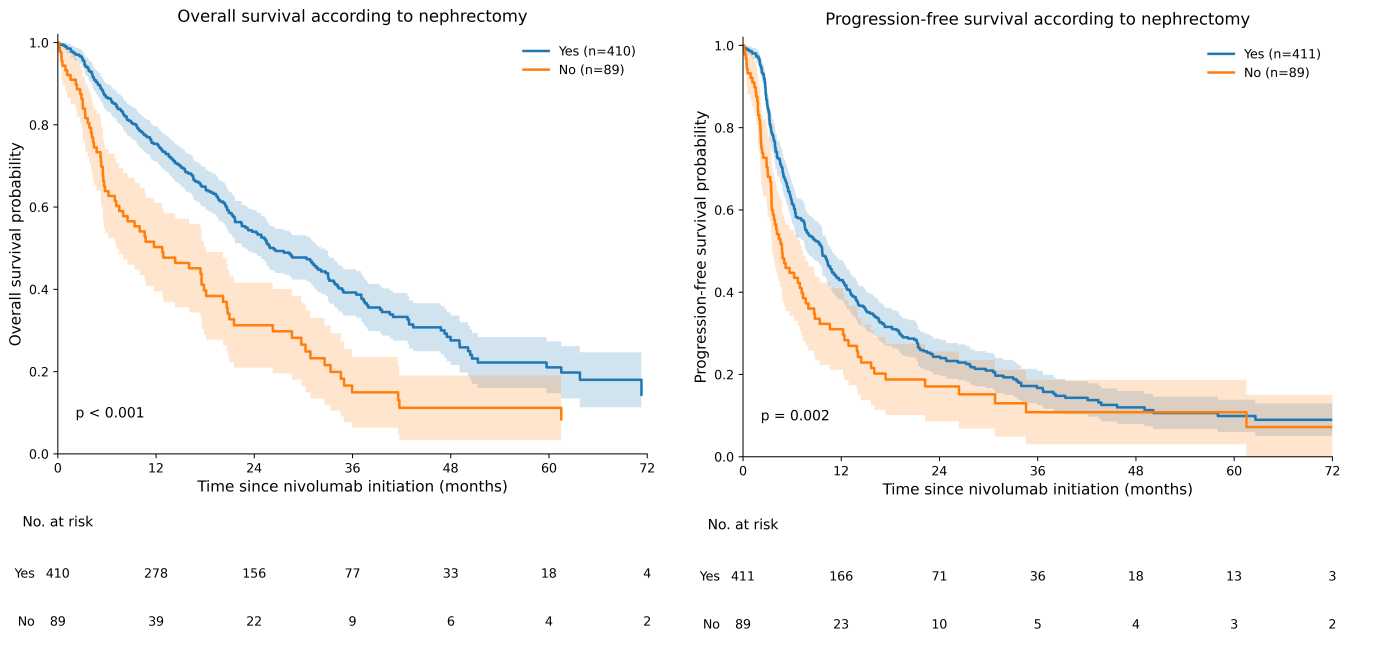


## Figure 1S. Overall survival (OS) and progression-free survival (PFS) according to previous nephrectomy.


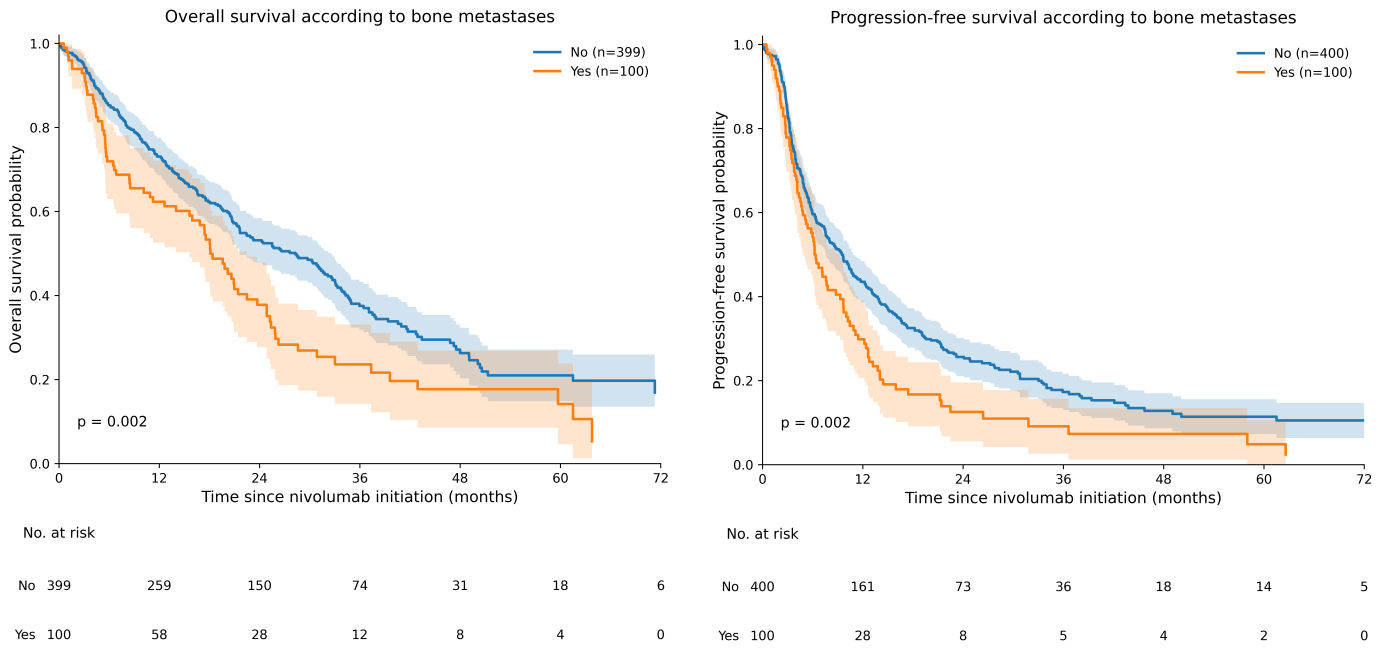


## Figure 2S. Overall survival (OS) and progression-free survival (PFS) according to bone metastases.


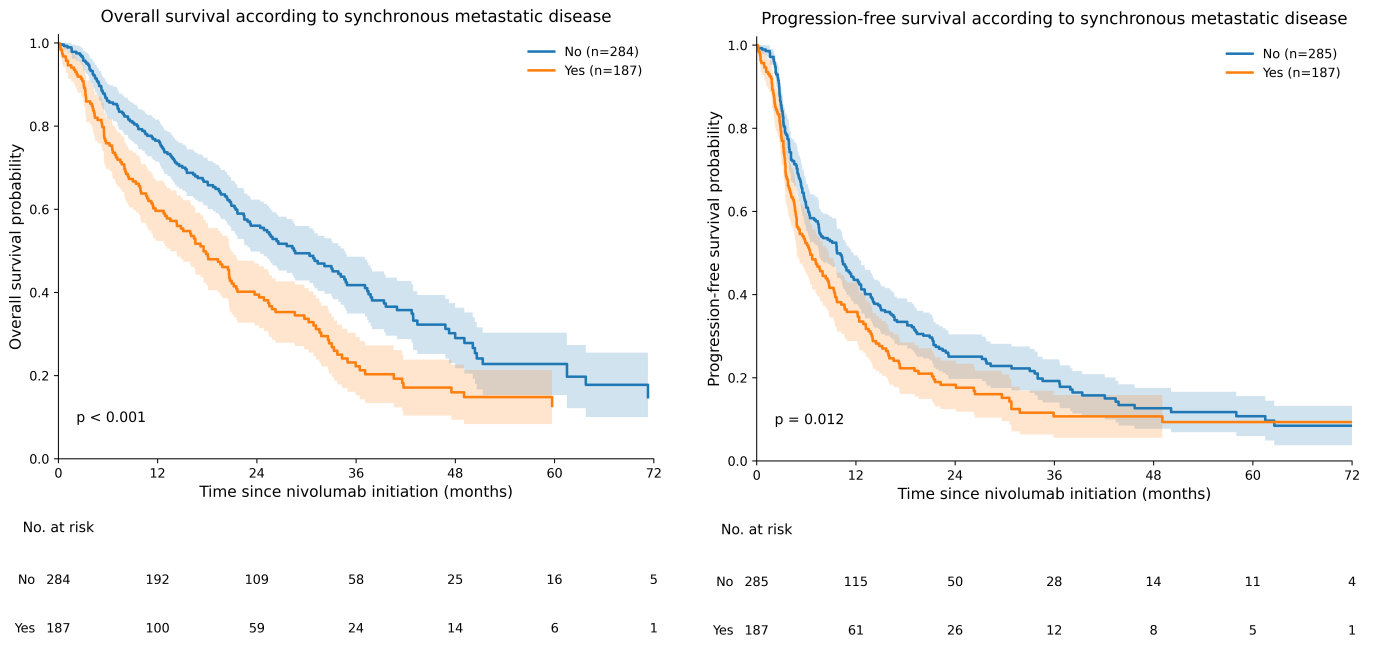


## Figure 3S. Overall survival (OS) and progression-free survival (PFS) according to synchronous metastatic disease.


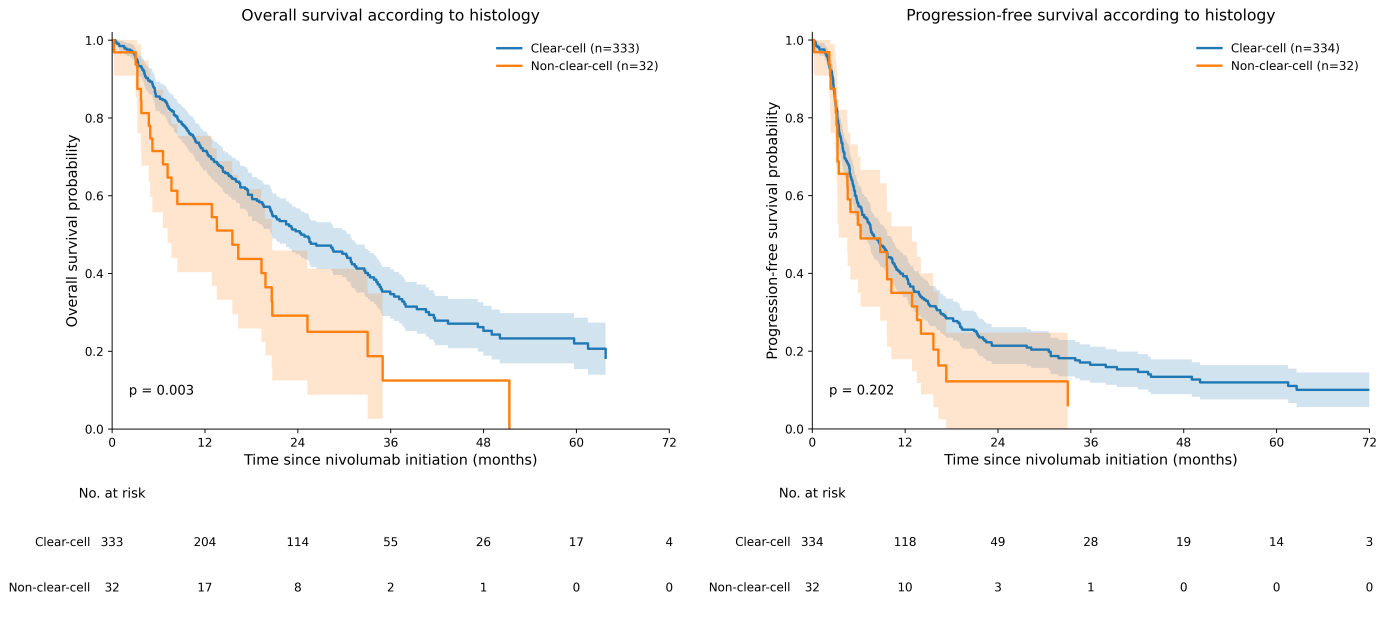


## Figure 4S. Overall survival (OS) and progression-free survival (PFS) according to histology.


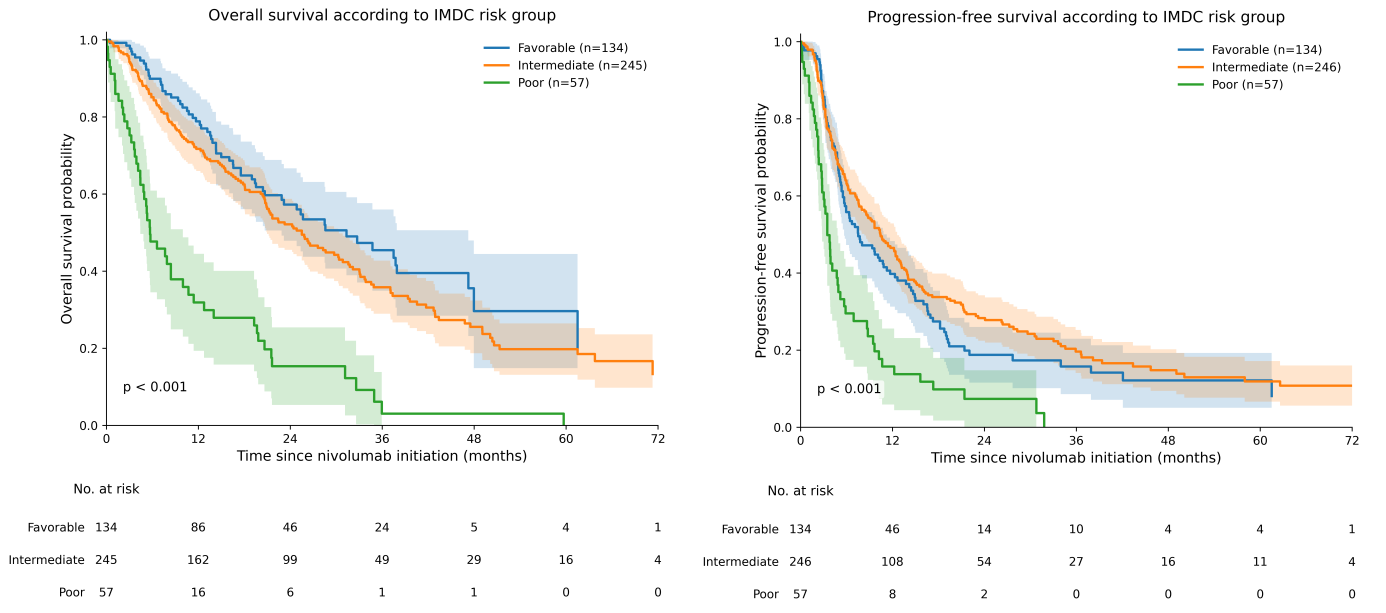


## Figure 5S. Overall survival (OS) and progression-free survival (PFS) according to IMDC risk group

##
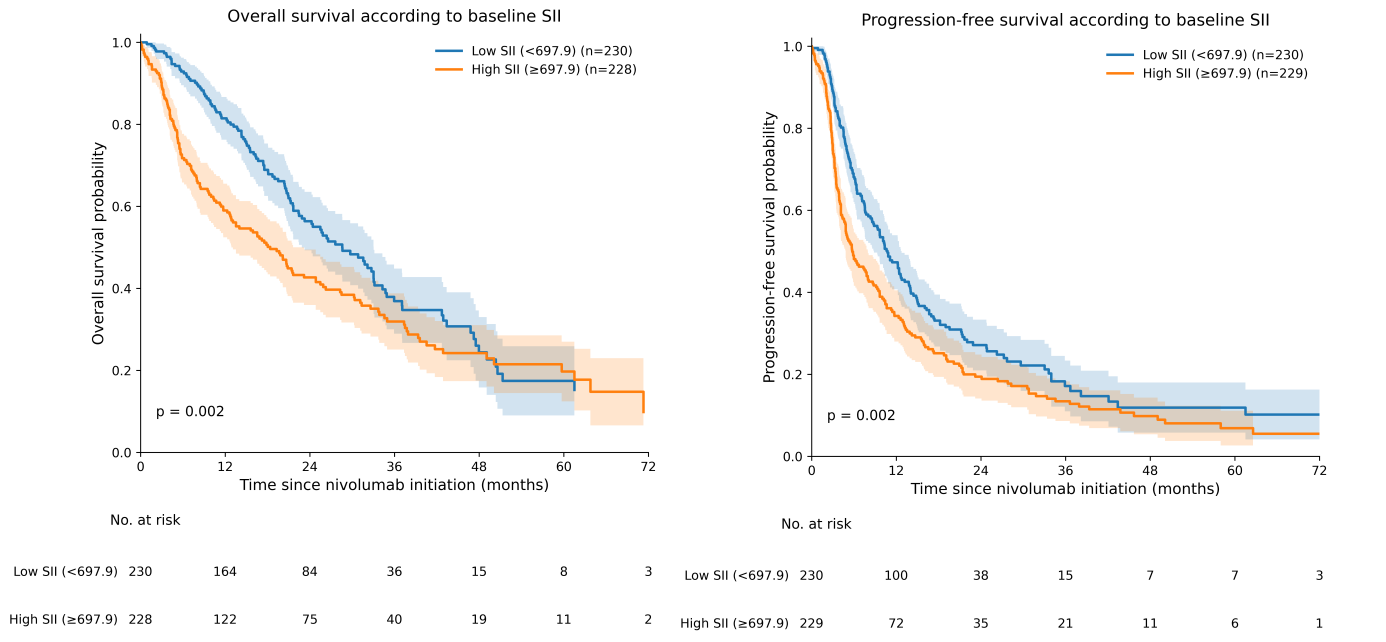
.

## Figure 6S. Overall survival (OS) and progression-free survival (PFS) according to baseline systemic inflammatory index (SII)

| **Variable** | **Overall survival (OS)** | | | **Progression-free survival (PFS)** | | |
| --- | --- | --- | --- | --- | --- | --- |
|  | **HR** | **95% CI** | **p value** | **PFS HR** | **95% CI** | **p value** |
| **Age** (per year) | 0.99 | 0.98–1.01 | 0.315 | 0.99 | 0.98–1.00 | 0.032 |
| **Male sex** | 1.07 | 0.79–1.44 | 0.676 | 0.79 | 0.62–1.02 | 0.066 |
| **ECOG PS** ≥2 vs 0–1 | 4.21 | 2.59–6.87 | <0.001 | 4.35 | 2.69–7.04 | <0.001 |
| **Line of therapy** 3L+ vs 2L | 1.10 | 0.84–1.45 | 0.482 | 1.05 | 0.83–1.34 | 0.662 |
| **Previous nephrectomy** no vs yes | 1.62 | 1.13–2.33 | 0.010 | 1.46 | 1.06–2.03 | 0.022 |
| **Bone metastases** yes vs no | 1.13 | 0.81–1.57 | 0.479 | 1.37 | 1.02–1.83 | 0.034 |
| **Synchronous metastatic disease** yes vs no | 1.05 | 0.77–1.44 | 0.750 | 1.01 | 0.78–1.31 | 0.950 |
| **Non-clear-cell vs clear-cell** | 1.02 | 0.60–1.72 | 0.950 | 0.75 | 0.45–1.23 | 0.255 |
| **Missing/unknown histology vs clear-cell** | 0.77 | 0.56–1.05 | 0.095 | 0.62 | 0.47–0.81 | <0.001 |
| **IMDC** poor vs favorable/intermediate | 2.21 | 1.45–3.38 | <0.001 | 1.65 | 1.11–2.45 | 0.014 |
| **High NLR vs low NLR** | **1.33** | **1.02–1.74** | **0.038** | **1.40** | **1.11–1.77** | **0.005** |

*Model sample size: N=395; OS events=247; PFS events=325. Baseline NLR was dichotomized at the cohort median.*

**Table 1S.** Multivariable Cox regression model for overall survival and progression-free survival including baseline neutrophil-to-lymphocyte ratio (NLR).

| **Variable** | **Overall survival (OS)** | | | **Progression-free survival (PFS)** | | |
| --- | --- | --- | --- | --- | --- | --- |
|  | **OS HR** | **95% CI** | **p value** | **PFS HR** | **95% CI** | **p value** |
| **Age** (per year) | 0.99 | 0.98–1.01 | 0.359 | 0.99 | 0.98–1.00 | 0.048 |
| **Male sex** | 1.09 | 0.81–1.47 | 0.586 | 0.81 | 0.63–1.04 | 0.102 |
| **ECOG** PS ≥2 vs 0–1 | 4.32 | 2.66–7.02 | <0.001 | 4.48 | 2.78–7.24 | <0.001 |
| **Line of therapy** 3L+ vs 2L | 1.14 | 0.87–1.50 | 0.338 | 1.07 | 0.84–1.36 | 0.579 |
| **Previous nephrectomy** no vs yes | 1.66 | 1.15–2.40 | 0.006 | 1.49 | 1.07–2.07 | 0.017 |
| **Bone metastases** yes vs no | 1.12 | 0.81–1.57 | 0.490 | 1.34 | 1.00–1.79 | 0.052 |
| **Synchronous metastatic disease** yes vs no | 1.06 | 0.78–1.44 | 0.712 | 1.02 | 0.78–1.32 | 0.900 |
| **Non-clear-cell vs clear-cell** | 1.09 | 0.65–1.83 | 0.735 | 0.81 | 0.50–1.33 | 0.406 |
| Missing/unknown histology vs clear-cell | 0.79 | 0.58–1.07 | 0.133 | 0.64 | 0.49–0.85 | 0.002 |
| **IMDC** poor vs favorable/intermediate | 1.97 | 1.27–3.03 | 0.002 | 1.53 | 1.03–2.28 | 0.037 |
| **High PLR vs low PLR** | **1.34** | **1.01–1.76** | **0.040** | **1.29** | **1.02–1.63** | **0.033** |

*Model sample size: N=395; OS events=247; PFS events=325. Baseline NLR was dichotomized at the cohort median.*

**Table 2S.** Multivariable Cox regression model for overall survival and progression-free survival including baseline platelet-to-lymphocyte ratio (PLR).

| **Variable** | **OS HR** | **95% CI** | **p value** | **PFS HR** | **95% CI** | **p value** |
| --- | --- | --- | --- | --- | --- | --- |
| **Age** (per year) | 0.99 | 0.98–1.01 | 0.313 | 0.99 | 0.98–1.00 | 0.039 |
| **Male sex** | 1.10 | 0.82–1.48 | 0.525 | 0.83 | 0.64–1.06 | 0.128 |
| **ECOG** PS ≥2 vs 0–1 | 4.42 | 2.71–7.21 | <0.001 | 4.47 | 2.76–7.23 | <0.001 |
| **Line of therapy** 3L+ vs 2L | 1.11 | 0.85–1.45 | 0.456 | 1.06 | 0.84–1.35 | 0.615 |
| **Previous nephrectomy** no vs yes | 1.65 | 1.15–2.37 | 0.007 | 1.48 | 1.07–2.05 | 0.018 |
| **Bone metastases** yes vs no | 1.14 | 0.82–1.59 | 0.437 | 1.36 | 1.01–1.82 | 0.040 |
| **Synchronous metastatic disease** yes vs no | 1.04 | 0.77–1.42 | 0.787 | 1.00 | 0.77–1.29 | 0.986 |
| **Non-clear-cell vs clear-cell** | 1.10 | 0.66–1.85 | 0.713 | 0.82 | 0.50–1.34 | 0.425 |
| Missing/unknown histology vs clear-cell | 0.80 | 0.59–1.09 | 0.159 | 0.65 | 0.49–0.85 | 0.002 |
| **IMDC** poor vs favorable/intermediate | 2.17 | 1.42–3.33 | <0.001 | 1.60 | 1.07–2.38 | 0.022 |
| **High SII vs low SII** | **1.10** | **0.84–1.44** | **0.483** | **1.21** | **0.96–1.52** | **0.115** |

*Model sample size: N=395; OS events=247; PFS events=325. Baseline NLR was dichotomized at the cohort median.*

**Table 3S.** Multivariable Cox regression model for overall survival and progression-free survival including baseline systemic immune-inflammation index (SII).
